# Intestinal and Extraintestinal Findings of Graft-versus-Host Disease on CT: A Case Series with Radiological and Histopathological Correlations

**DOI:** 10.3390/biomedicines12071516

**Published:** 2024-07-08

**Authors:** Barbara Brogna, Camilla Frieri, Antonio Maria Risitiano, Luigi Urciuoli, Gabriella Storti, Lidia Santoro, Eleonora Urciuoli, Giovanni De Chiara, Pasquale Cretella, Carmen Sementa, Lanfranco Aquilino Musto, Francesca Maccioni

**Affiliations:** 1 Unit Interventional and Emergency Radiology, AORN, San Giuseppe Moscati Hospital, Contrada Amoretta, 83100 Avellino, Italy; luigiurciuoli@yahoo.it (L.U.); musto.lanfranco@gmail.com (L.A.M.); 2 Hematology and Bone Marrow Transplant Unit, AORN, San Giuseppe Moscati Hospital, Contrada Amoretta, 83100 Avellino, Italy; camillafrieri@gmail.com (C.F.); amrisita@unina.it (A.M.R.); stortigabriella2@gmail.com (G.S.); lidiasantoro63@gmail.com (L.S.); ele.urc@gmail.com (E.U.); 3 Division of Pathologic Anatomy, AORN San Giuseppe Moscati Hospital, Contrada Amoretta, 83100 Avellino, Italy; endocrinologiadechiara@gmail.com (G.D.C.); pasquale.cretella@aornmoscati.it (P.C.); 4 Forensic Medicine Unit, AORN San Giuseppe Moscati Hospital, Contrada Amoretta, 83100 Avellino, Italy; carmensementa1@gmail.com; 5 Department of Radiological, Oncological and Pathological Science, Umberto I Hospital, La Sapienza University of Rome, Viale Regina Elena 324, 00161 Rome, Italy; francesca.maccioni@uniroma1.it

**Keywords:** acute GVHD, chronic GVHD, gastrointestinal GVHD, contrast-enhanced computed tomography, intestinal bowed disease

## Abstract

Graft-versus-host disease (GVHD) is an expected and relatively common complication after allogeneic hematopoietic stem cell transplantation. It may affect different organs and typically involves the skin, liver, and gastrointestinal tract (GI-GVHD). GI-GVHD may show heterogeneous presentations with peculiar diagnostic implications. Although an endoscopic biopsy is considered the “gold standard” for the diagnosis of GI-GVHD, its broad application is limited due to the poor clinical conditions usually present in these patients, including thrombocytopenia. In the emergency department, enhanced computed tomography (CECT) has emerged as the best imaging modality for the evaluation of GI damage in frail patients. However, the role of CT in the context of either acute or chronic GI-GVHD has not been systematically investigated. Herein, we focus on the radiological features found on CECT in five patients with GI-GVHD confirmed on histology. CECT was performed for the persistence of GI symptoms in three cases (case 1, case 3, and case 4), for small bowel occlusion in one case (case 5), and for acute GI symptoms in one case (case 2). Serpiginous intestinal wall appearance with multisegmental parietal thickness and homogeneous, mucosal, or stratified small bowel enhancement were common features. Colic involvement with segmental or diffuse parietal thickness was also present. One patient (case 5) presented with inflammatory jejunal multisegmental stenosis with sub-occlusion as a chronic presentation of GI-GVHD. Regarding mesenterial findings, all five patients presented comb signs in the absence of lymphadenopathy. Extraintestinal findings included biliary tract dilatation in two cases (case 2 and case 4). These data support the utility of appropriate radiological investigation in GI-GVHD, paving the way for further serial and systematic investigations to track the appearance and evolution of GI damage in GVHD patients.

## 1. Introduction

Graft-versus-host disease (GVHD), although rare, is an immunological disorder that can frequently occur after allogeneic hematopoietic stem cell transplantation (HSCT). It is caused by the immune reaction of the donor immunocompetent T cells, which may derive directly from the graft and/or from newly generated lymphoid cells and may typically recognize antigens of either the major or minor histocompatibility complex (MHC) of the recipient [1,2]. The incidence of GVHD increases with HLA disparity, being lower with HLA-matched sibling donors and higher with HLA-matched unrelated donors, and even more prevalent with mismatched donors. Among the minor antigens, Y chromosome-associated antigens may also contribute, accounting for higher GVHD rates in sex mismatching [1,2,3]. GVHD is conventionally split into two main clinical forms: the acute form (aGVHD), when symptoms begin within the first 100 days after the transplant procedure, and the chronic form (cGVHD), when symptoms begin after day 100 [1,2,3]. However, the emergence of broader strategies of HSCT (e.g., non-myeloablative conditioning, in vivo T-cell depletion, HSCT across the HLA barrier) has been challenged by the recognition that signs of acute and chronic GVHD may occur outside of these designated periods. The current classifications distinguish the two forms based on the clinical presentations along the three typical target organs: skin, liver, and GI tract [1,2,3,4,5].

Briefly, aGVHD usually manifests with erythematous or maculopapular rash, nausea with vomiting or diarrhea, and cholestatic hepatitis. It can be classified as classical acute until day 100 and beyond day 100 as late-onset, persistent, or recurrent aGVHD (features of acute GVHD occurring after 100 days in the absence of chronic GVHD) [2,4]. Similarly, cGVHD is subdivided as classic or overlap syndrome, the latter having coexisting acute and chronic features [2,6,7].

Acute GVHD is typically graded according to the modified Glucksberg scale [8,9,10,11]. Each organ system is staged individually according to severity, and then an overall grade is assigned based on the severity of each organ system. However, it is often very difficult to distinguish the characteristic features of aGVHD from those of other complications, such as general drug toxicity and infection, and, consequently, to determine the appropriate choice of treatment [4]. For this reason, it is essential to establish the diagnosis via biopsy of one or more affected organs to confirm the characteristic histopathological features [4,11,12,13]. The targets of the immune response in aGVHD are the epithelial cells (including basal and suprabasal cells of the epidermis), the intestinal epithelium, and the biliary duct epithelium. The characteristic feature of the response is identical in each case and consists of the presence of infiltrating immune cells close to apoptotic cells, known as “satellite cell necrosis” [4,11,12]. More specifically, in aGVHD, the most important pathologic event is a combination of enterocyte apoptosis, basal gland destruction (“crypt apoptosis”), and mucosal denudation. In mild and early cases of aGVHD, crypt apoptosis is used as a single diagnostic criterion [12]. Chronic inflammatory infiltrate, which is denser around injured crypts, may also be found. The presence of granulocytes, eosinophils, and a loss of crypt Paneth cells correlate with a poor prognosis [12].

On the other hand, the diagnosis of cGVHD is based on symptoms and laboratory values [5,12,13,14]. Chronic GVHD can imitate almost any autoimmune disease and can affect a number of organs, and patients often do not report changes until functional impairment is recognized [5,13,14]. Histologically, findings such as gland architectural distortion (i.e., cystic gland formation) and/or destruction, ulceration, or submucosal fibrosis reflect long-standing disease but are not specific for cGVHD [15,16,17]. The gastrointestinal (GI) tract is one of the most frequent nonlymphoid organ sites involved in aGVHD [11,12].

The characteristic clinical manifestations of GI aGVHD are secretory and voluminous diarrhea, nausea, vomiting, distention, and paralytic ileus [4,11,12]. However, the GI tract is less commonly affected by cGVHD [5,12]. In such cases, the clinical manifestations are generally chronic diarrhea and weight loss [5,12].

The chronic form, being characterized by fibrosis [15,16,17], can manifest itself with stenosis and subocclusive symptoms [18,19,20,21,22,23,24].

GVHD affects the GI tract heterogeneously, and although endoscopic biopsy is considered the “gold standard” for the diagnosis of GI-GVHD, numerous limitations hamper its broad application in daily practice. For instance, its diagnostic sensitivity and specificity are far from ideal, and up to 26% of patients receive treatment for a possible GI-GVHD despite a negative endoscopic biopsy [25]. This may also be due to the fact that GI damage is heterogeneous, and sampling cannot always be driven by endoscopy since some locations remain inaccessible to endoscopic investigations (e.g., the small intestine); histological changes may even be found in macroscopically normal areas [11,12,15,25,26]. In addition, biopsy samples may be inadequate, and sometimes even the pathology description is not conclusive due to the lack of specificity of some histological findings, which often require a complex differential diagnosis with other clinical conditions [11,12,15,25,26]. Furthermore, endoscopy still represents a relatively invasive diagnostic procedure that can be partially or absolutely contraindicated in patients suffering from severe thrombocytopenia, granulopenia, and/or coagulopathy, such as HSCT recipients [25,26,27,28].

As a consequence, in daily practice, clinical parameters remain the key element to be considered for the initial suspect and possible diagnosis of GVHD. In this context, the most common clinical parameter is the daily volume of diarrhea, which is obviously inconvenient and imprecise [4,11,25,27]. However, abdominal contrast-enhanced computed tomography (CECT) is the best imaging modality for use during emergencies in frail patients requiring HSCT. This is because CECT has benefits such as speed, high spatial and temporal resolution, ease of accessibility, and the possibility of using multiplanar reconstruction. In fact, CT may play a pivotal role in supporting the clinical diagnosis of GI GVHD, with the potential advantage of non-invasive evaluation of the GI tract, including endoscopically inaccessible regions [25,26,27,28,29,30,31,32,33,34]. It is also possible to use CT to evaluate the possible origin of abdominal sepsis, which is often a frequent complication in these frail patients [26,30,32].

Moreover, some studies have demonstrated how the abdominal findings detected on CT correlate with the clinical and pathological grade [31,33].

However, GI-GVHD remains a diagnostic challenge, and radiological findings are often poorly understood by radiologists themselves. The findings of intestinal aGVHD are similar to those observed in any inflammatory or infectious enteritis [26,28,29,30,31,32,33]. However, there are few imaging studies on cGVHD, and only case reports have been published [18,19,20,21,22,23,24].

Herein, we present five cases of histologically confirmed GI-GVHD focusing on the radiological CECT features, aiming to identify radiological findings that appear to be recurrent in specific clinical conditions.

## 2. Case Presentations

### 2.1. Case 1

**Figure 1:**
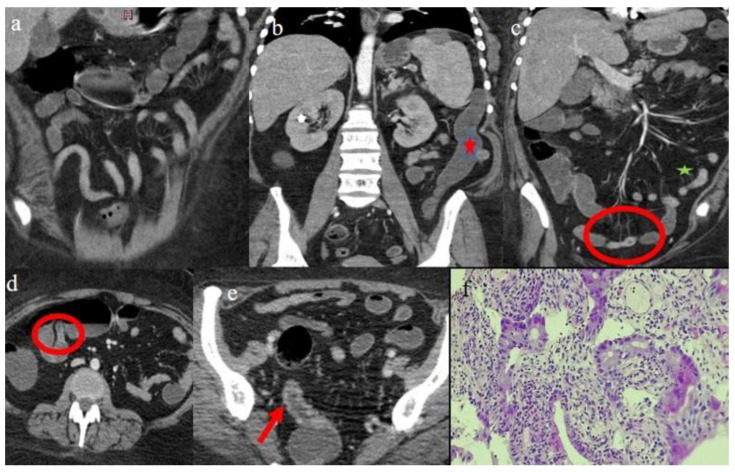
Image findings of GI aGVHD on the CECT of case #1. Image (**a**) shows the serpiginous appearance of the intestinal loops and image (**b**) (red arrow) shows the tubular appearance of the descending colon (red star) and a mild comb sign (green star). Images (**c**,**d**), respectively, show a segmental and focal thickness with homogeneous and mucosal enhancement at the level of distal ileus (red circle). Image (**e**) shows a mild segmental parietal thickness at the level of the sigma with homogeneous enhancement (red arrow). Image (**f**) shows colic mucosa with distorted crypts, areas of crypt apoptosis, and sparse granulocytic inflammation, with associated cryptitis. Hematoxylin and eosin staining, 100×.

Patient #1 was a 28-year-old woman with a previous history of acute myeloid leukemia (AML). She underwent HSCT from a haploidentical donor, receiving a conditioning regimen based on thiotepa, busulfan, and fludarabine. A GVHD prophylaxis was made using post-transplant cyclophosphamide (PT-Cy), cyclosporine (CsA), and mycophenolate mofetil (MMF). She already showed mild signs of cutaneous GVHD on day 35 post-transplant; however, the clinical course worsened about 5 months later due to the suspension of immunosuppressive therapy with GI involvement (Table 1). For this reason, she underwent a CECT, performed 155 days (about 5 months) after the HSCT. The CECT was requested for the persistence of diarrhea and abdominal pain. The CECT showed a serpiginous appearance “ribbon sign” of the small bowel loops (Figure 1a), a mild colic distension with a tubular appearance with a loss of haustral folds of the descending colon (Figure 1b), and a mild fluid distension of the terminal ileus (Figure 1c). Also present on CECT were a multisegmental and focal thickness of the distal ileus, with homogeneous and mucosal enhancement of some loops (Figure 1c,d), and a multisegmental thickness of the sigma (Figure 1e). The intestinal loops showed diffuse, mild mesenteric congestion “comb sign” and poor lymph nodes (Figure 1a,c). As an extraintestinal finding, this patient presented a small hypodense area on the superior pole of the spleen as a splenic infarct (Figure 1b) (Table 2). The histologic diagnosis made on biopsy fragments of ileal and colon mucosa was consistent with persistence of aGVHD, showing an intense, chronic active inflammatory infiltrate of the lamina propria and a reduction in crypts, with images of apoptotic necrosis, cryptitis, and focal formation of cryptic microabscesses. Regenerative hyperplasia of the epithelium was also present.

### 2.2. Case 2

**Figure 2:**
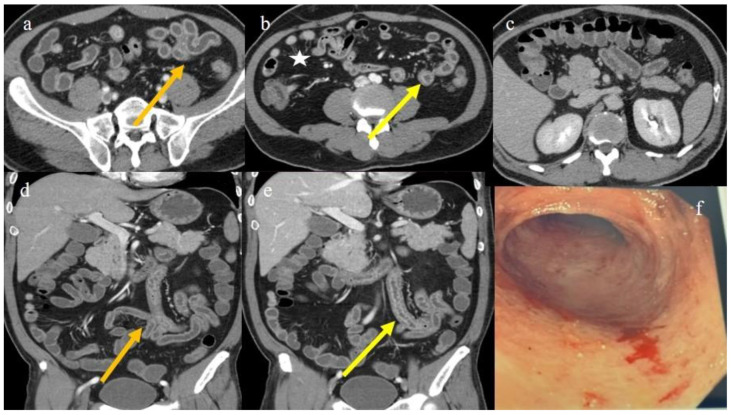
The CECT findings of GI aGVHD in case #2. Image (**a**) (orange arrow) shows a serpiginous appearance of the intestinal loops with homogeneous mucosal enhancement on the axial plane. Image (**b**) (yellow arrow) shows the stratified enhancement of some jejuna loops with mild comb sign white star). Image (**c**) shows the fluid stasis in the colon. Image (**d**) shows a serpiginous appearance (orange arrow) of the jejuna loops on the coronal plane. Image (**e**) shows the stratified enhancement of the jejuna loops on the coronal plane. Image (**f**) shows the endoscopic findings of ileal colonoscopy, showing a superficial lesion of the mucosa with bleeding.

A 51-year-old man with a previous history of Hodgkin lymphoma (HL) underwent HSCT from a haploidentical donor, receiving a conditioning regimen based on thiotepa, busulfan, and fludarabine. A GVHD prophylaxis was made using PT-Cy, cyclosporine, and mycophenolate mofetil. On day 20 after HSCT, he presented with profuse diarrhea, and a clinical diagnosis of GVHD with GI intestinal involvement was made (Table 1). The CECT was requested for acute abdominal pain and showed a ribbon-like appearance (Figure 2a,d) of the intestinal loops with diffuse parietal thickness, homogeneous enhancement of the small bowel loops, and stratified enhancement of some jejunal loops (Figure 2b,e). A mild colic and small bowel fluid distension (Figure 2c) were present. Diffuse mesenteric congestion with a comb sign was also present with poor reactive lymph nodes (Figure 2b,e). A mild thickness of the stomach walls at the level of the antropyloric region was described on CECT. The extraintestinal findings included a mild distension of the biliary tree and a mild gallbladder thickness.

The ileal colonoscopy showed superficial lesions of the mucosa with easy bleeding alternated with normal mucosa (Figure 2f).

The histological findings were consistent with the diagnosis of GVHD with widespread areas of ulceration, with active granulocytic inflammation and widespread atrophy of the cryptic glandular component, replaced by inflammatory granulation tissue with neoangiogenesis.

### 2.3. Case 3

**Figure 3:**
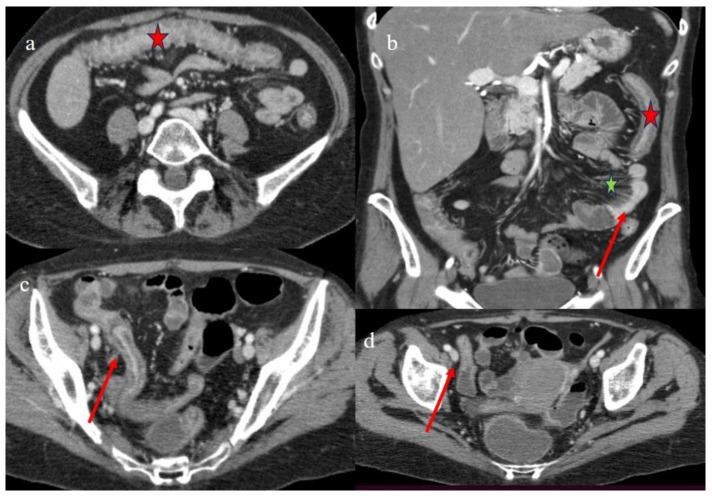
The CECT findings of the GI aGVHD in case #3, 92 days after HSCT. The diffuse colic homogeneous thickness with stratified enhancement (red star) is shown on the axial plane in image (**a**) and on the coronal plane in image (**b**). Image (**b**) also shows a segmental small bowel thickness with homogeneous enhancement (red arrow) with diffuse comb sign (green star). Image (**c**) shows the stratified enhancement of the terminal ileus (red arrow) with mild fluid distension at the level of the rectus and sigma. Image (**d**) shows the appendix thickness (red arrow).

Patient #3 was a 28-year-old woman with a diagnosis of acute lymphoblastic leukemia Ph+ (ALL) who underwent HSCT from an HLA sibling donor. The conditioning regimen was based on TBI (total body irradiation) and fludarabine. A GVHD prophylaxis was made using cyclosporine (CsA) and methotrexate (MTX). For clinical manifestations of GI-GVHD (Table 1), she underwent a CECT 92 days (about 3 months) after transplant. On CECT, the patient showed a diffuse colic thickness with stratified enhancement (Figure 3a,b) and a mild fluid distension at the level of the rectus and sigma (Figure 3c,d). A multisegmental intestinal wall thickness with a stratified enhancement on the terminal ileus was also present (Figure 3b,c), with appendiceal involvement (Figure 3d). There was also a diffuse mesenteric congestion “comb sign” with poor reactive lymph nodes (Figure 3b). Some intestinal loops had a serpiginous appearance. The extraintestinal findings are summarized in Table 2.

Another CECT was requested on day 129 post-HSCT due to the persistence of the abdominal symptoms.

**Figure 4:**
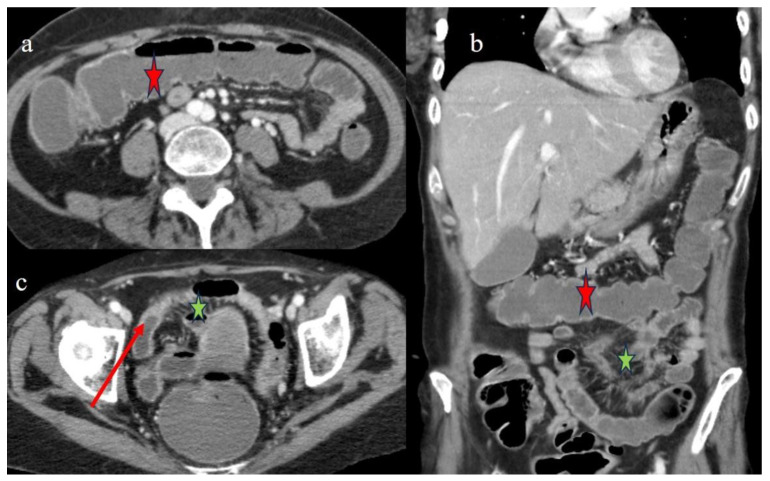
The CECT findings of case #3 129 days after HSCT. Images (**a**,**b**) show the colic fluid distension (red star) with mucosal enhancement on the axial and coronal plane, respectively. Image “c” shows the persistence of terminal ileal thickness with mucosal enhancement (red arrow). The diffuse comb sign is shown in images (**b**,**c**) (green star).

The CECT showed a homogeneous fluid distension of the colon and of some small bowel loops, with a reduced parietal thickness of the colic walls but diffuse mucosal enhancement (Figure 4a,b). The intestinal loops showed the persistence of the parietal thickness in the distal ileus, but with a reduction compared to the previous control (Figure 4c). The “comb sign” continued to be present (Figure 4b,c).

**Figure 5:**
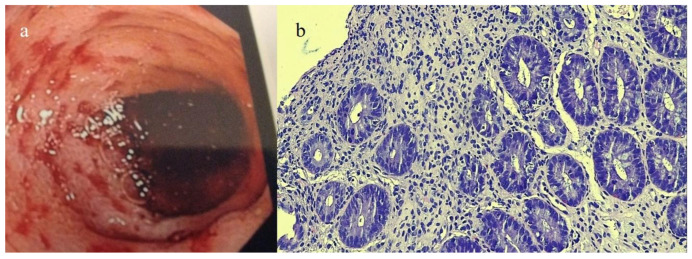
Image (**a**) shows the colonoscopy findings with a superficial lesion of the mucosa with bleeding, and image (**b**) shows colic mucosa with increased crypt apoptosis in case #3. Hematoxylin and eosin staining, 100×.

The ileal colonoscopy showed easily bleeding superficial lesions of the mucosa (Figure 5a). The histology of the biopsy samples confirmed the diagnosis of acute GVHD of the intestinal mucosa with the presence of perifocal phenomena of cryptic apoptosis, with slight thinning of the glandular crypts (drop-out) and the presence of some atrophic crypts (Figure 5b).

### 2.4. Case 4

**Figure 6:**
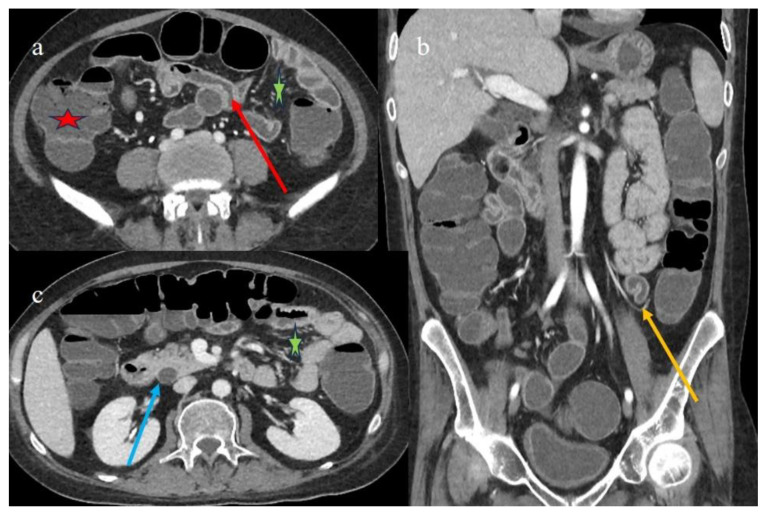
The CECT features on day 66 after HSCT in case #4. Image (**a**) represents the mild parietal thickness of some small bowel loops with homogeneous mucosal enhancement (red arrow), a mild colic distension with air-fluid level (red star), and a mild comb sign (green star). Image (**b**) shows the serpiginous appearance of some intestinal loops (orange arrow) and the colic fluid distension. The main biliary duct distension is shown in image (**c**) (blue arrow).

A 33-year-old man with a previous history of severe aplastic anemia (SAA) received a transplant from his HLA sibling donor, followed by a conditioning regimen based on serum anti-T lymphocyte globulin (ATG), fludarabine, and cyclophosphamide. A GVHD prophylaxis was made using cyclosporine. He developed signs of GI-GVHD and underwent a CECT on day 66 (about 2 months) after HSCT (Table 1). On CECT, the patient showed mild intestinal and colic distension with air-fluid level (Figure 6) and a multisegmental homogeneous small bowel mucosal enhancement with mild parietal thickness. The ribbon sign appearance of the intestinal wall was present, as was mesenteric vessel congestion. The patient also showed mild parietal thickness of the second portion of the duodenum and moderate distension of the main bile duct.

**Figure 7:**
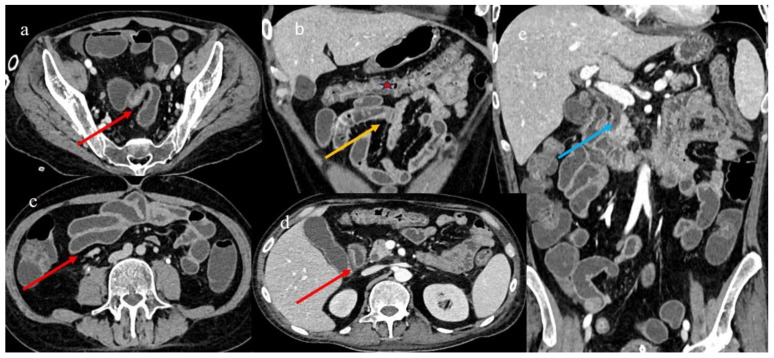
Image (**a**) represents a segmental thickness with mucosal enhancement at the level of the sigma (red arrow) on day 120 after the HSCT of case #4. Images (**b**,**c**) show that the serpiginous appearance (orange arrow) and the parietal thickness with mucosal enhancement (red arrow) were more evident than in the previous control. Image (**b**) also shows the stratified enhancement of the transverse colon (red star). Image (**d**) shows the duodenal thickness (red arrow), and image (**e**) shows the persistence dilatation of the main biliary duct (blue arrow).

The CECT, performed on day 120 after the HSCT, continued to show mild fluid colic distension with the appearance of multisegmental thickness in the sigma and descending colon and with mucosal enhancement and stratified enhancement on the transverse colon (Figure 7a,b). The intestinal loops were more involved than on the baseline CT examination and presented with homogeneous diffuse parietal thickness and homogeneous mucosal enhancement (Figure 7c). The ribbon sign appearance was more prominent (Figure 7b), as was the parietal thickness of the second portion of the duodenum (Figure 7e). The main bile duct continued to be distended (Figure 7e). The extraintestinal findings are summarized in Table 2.

**Figure 8:**
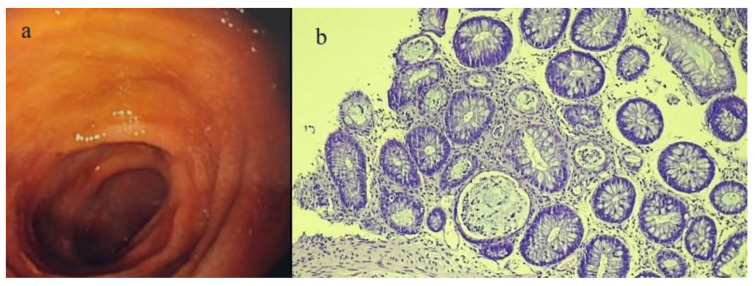
Image (**a**) represents the colonoscopy findings of edema and loss of vascular pattern, and image (**b**) shows colic mucosa with extensive cryptic apoptosis, intraluminal apoptotic debris, and focal atrophic crypts in case #4. Hematoxylin and eosin staining, 100×.

On colonoscopy, the patient showed an edematous appearance of the mucosa with a loss of vascular representation (Figure 8a).

The morphological features on histology were consistent with aGVHD and were characterized by the widespread presence of cryptic apoptosis with intraluminal apoptotic debris and the presence of some atrophic crypts, in the absence of significant thinning (drop-out) of the crypts. Mild granulocytic inflammation with instances of cryptitis was also present (Figure 8b).

**Figure 9:**
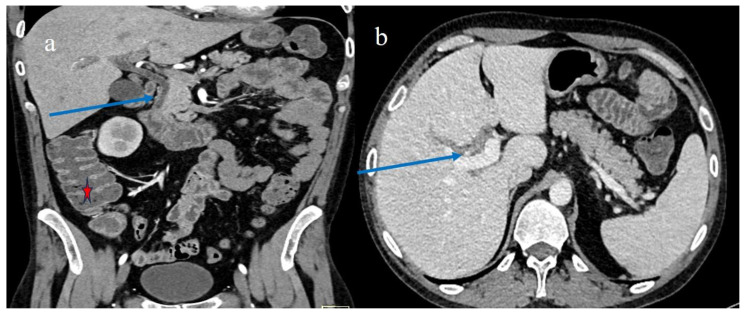
The CECT control for case #4 after 8 months showed a mild fluid colic distension (image (**a**), red star) with the persistence of dilatation of the main biliary duct (image (**a**,**b**) blue arrow).

A CECT control was also made after eight months. It showed a mild colic distension and a persistent distension of the main bile duct (Figure 9).

### 2.5. Case 5

**Figure 10:**
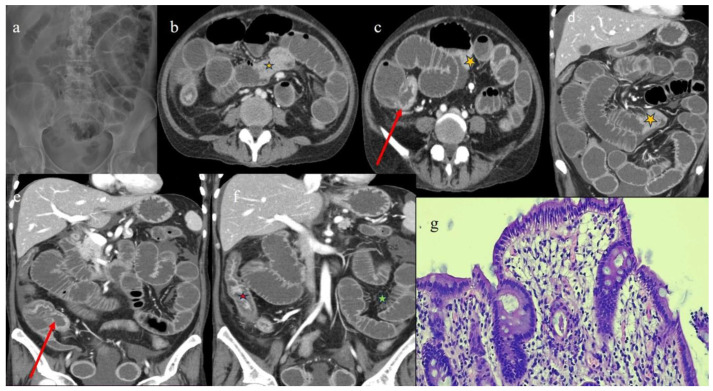
The CECT features seen in the emergency department 9 months after HSCT in case #5. Image (**a**) represents the CT scout view showing the serpiginous appearance of some small bowel loops. Images (**b**,**c,d**) show the long segmental inflammatory jejuna stenosis (orange star) with sign of SBO. A segmental and focal thickness of the terminal ileus and the appendix is also reported in images (**b**) (red arrow) and (**e**). Image (**f**) shows a diffuse stratified enhancement of the colon (red star) with mild comb sign (green star). Image (**g**) shows colic mucosa with architectural distortion of the glandular component, with associated mucin depletion and chronic inflammation of the lamina propria. Hematoxylin and eosin staining, 100×.

A 47-year-old woman with a diagnosis of acute lymphoblastic leukemia Ph+ (ALL) underwent a match-unrelated transplant, receiving a conditioning regimen based on TBI (total body irradiation) and fludarabine. A GVHD prophylaxis was made using cyclosporine (CsA) and methotrexate (MTX) (Table 1). Nine months after the transplant, she exhibited diarrhea, hematochezia, and persistent abdominal pain. She had a history of weight loss and malabsorption. She had no previous history of GVHD. Blood culture, urine culture, blood Epstein–Barr virus (EBV) and cytomegalovirus (CMV) in PCR-RT, and a nasopharyngeal aspiration respiratory viral study were all negative. A colonoscopy was performed, and histology on the biopsy sample was suggestive of chronic colitis in the chronic phase of GVHD (Figure 10). Fragments of the colon mucosa showed an architectural distortion of the glandular component (associated with a slight depletion of the mucosal portion), edema, and a mild chronic inflammatory infiltrate in the lamina propria with focal images of cryptitis up to occasional cryptic microabscesses (Figure 10). However, after a few days, her clinical symptoms worsened. A CECT was requested to rule out an abdominal occlusion. The CECT showed a serpiginous appearance of the small bowel loops, with multisegmental stenotic parietal thickness at the level of the jejunal–ileal loops (Figure 10). It also showed diffuse and marked distension of the rest of the intestine with signs of SBO. Other segmental and focal parietal thicknesses were also noted at the terminal ileus and the appendix, and a diffuse parietal thickness of the colon with stratified enhancement was also present (Figure 10). Stenosis in chronic GI-GVHD was suspected. A mild comb sign with mild ascites was also present. In this specific case of cGVHD, the CT signs of chronic ileal inflammation were very similar to those observed in Crohn’s disease.

**Figure 11:**
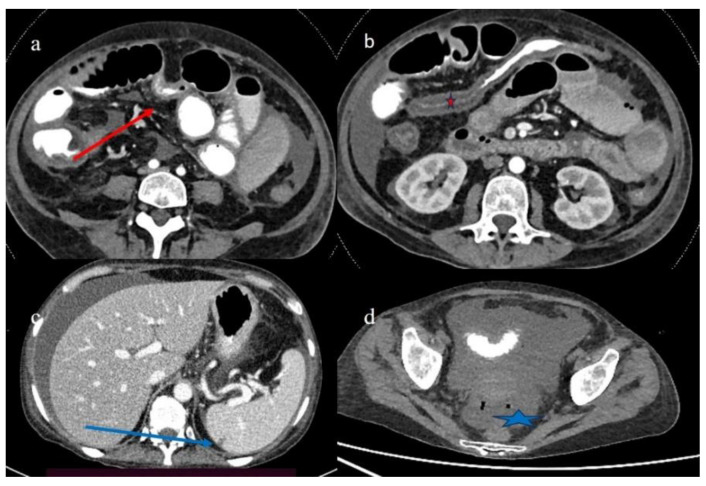
The CECT features made with oral contrast (gastrographin administration) a few days after the previous examination in case #5. Image (**a**) shows the mild resolution of the previous jejuna stenotic segmental stenosis (red arrow). Image (**b**) shows the stratified enhancement of the colon. Image (**c**) shows the small hypodense area in the spleen (blue arrow), and image (**d**) shows the free air bubble in the ascites (blue arrow).

A CECT control made a few days later showed a mild resolution of the previous intestinal stenosis (Figure 11a,b); however, small hypodense areas appeared in the spleen and some small air bubbles appeared in the pelvic ascites (Figure 11c,d), compatible with micro perforation. All the intestinal and extraintestinal findings are summarized in Table 2. Surgery was not performed. There was a high risk of mortality due to severe thrombocytopenia, and the patient subsequently died.

## 3. Discussion

This small case series confirms that the GI presentation of GVHD can be a pleomorphic and heterogeneous syndrome. The findings detected on CT in aGVHD can be found well beyond the classic temporal classification of 100 days, consistent with the concept that, once developed, organ damage from GVHD may take weeks or months to resolve (if it does), despite immunosuppressive treatments. Our cases 1, 3, and 4 showed CECT alterations at 4–5 months (120–155 days) after the allograft. Unlike case 5, which began with stenosis and sub-occlusion (more typical of chronic forms), cases 1, 3, and 4 were probably persistent forms of aGVHD. All our patients also showed a ribbon-like appearance of the intestinal loops, which would appear to be a characteristic feature of this disease. This appearance was found in all five of our cases, which included the acute forms (cases 2–4) and the persistent forms (cases 1, 3, and 4), as well as case 5, who presented in emergency with SBO. This finding could correlate with the endoscopic and histological aspects of denudation and desquamation of the mucosa, which, in the most severe cases, is accompanied by atrophy of the intestinal villi and loss of the crypts [11,12,35,36]. Furthermore, mucosal enhancement was common because the intestinal mucosa is the primary site of the inflammatory phlogistic process [11,35,36,37], consistent with the classical triad of GVHD, as proposed by Ferrara et al. [37]. During the conditioning regimen (irradiation, chemotherapy, or both), there is already damage to the intestinal barrier through the release of the inflammatory cytokines TNF-α and IL-1. This barrier loss may contribute to the prevention of alterations in the gut microbiome in GVHD and may amplify the damage and inflammatory response [11,36,37,38,39,40,41]. The impairment of intestinal barrier function that occurs in GVHD and associated dysbiosis are also characteristics of inflammatory bowel disease (IBD) [36,38,39,40]. Indeed, there is significant clinical, pathological, and genetic overlap between GVHD and IBD [40].

Immune dysregulation in aGVHD may result in substantial damage to the GI wall, with functional impairment of the intestinal barrier leading to failure of fluid reabsorption, particularly in the ileum, which is largely responsible for the voluminous diarrhea experienced by these patients [11,12,35,36]. In our patients, fluid stasis with slight distension of the intestinal loops, associated with distension of the colon, was a fairly frequent finding. These findings reflect the inflammatory phenomena of the acute phase and a reflex ileus. Thickening of the intestinal loops, of a multisegmental or diffuse type, was also frequent. These results are in line with previous studies [28,29,30,31,32,33,34]. Target enhancement with submucosal edema with “target” or “halo sign”, which is another characteristic sign of this disease, was detected in our case series in two cases affecting the intestinal loops (case 2 and case 3) and in three cases at the colonic level (case 3, case 4, and case 5). The presence of stratified enhancement may be correlated not only with the inflammation and barrier damage of the intestinal epithelium but also with the suffering of the vascular endothelium that occurs in these patients [41,42]. Case 5, who presented in an emergency with occlusive symptoms, was female and was the only one to have received stem cells from an unrelated donor. In this case, there was no previous history of acute GVHD. Approximately 30% of chronic GVHD cases can arise de novo without any previous acute GVHD [13]. In these cases, sub-stenotic thickening reflects the fibrotic thickening characteristic of chronic forms [30], and imaging with CT can explore both the parietal and extraparietal involvement that cannot be visualized by means of endoscopy, as in IBD. Rectal biopsy is known to have diagnostic utility in the diagnosis of aGVHD, using the presence of epithelial single-cell necrosis (apoptosis) as the main histological diagnostic criterion. This may be accompanied by increased inflammation (cryptitis) and reactive epithelial changes or loss [16].

However, there is no established role for endoscopic evaluation and mucosal biopsy in the diagnosis of GI cGVHD [16,17]. The histologic changes in chronic GI-GVHD (i.e., fibrosis, mononuclear cell infiltration, sclerosis, and hyalinization of small venules) are mainly confined to the submucosa and subserosa and may not be captured by the biopsy sample. Additional features suggestive of GI cGVHD on histology are significant mucosal architectural (crypt) distortion, increased lymphocyte/plasma cell infiltration in the lamina propria, and the presence of Paneth cell metaplasia (defined by Paneth cells in the left colon and/or rectum, which are normally present in the right and transverse colon) [15,16,17]. Nevertheless, as reported in the NIH chronic GVHD Diagnosis and Staging consensus manuscript, biopsies can be necessary to confirm the diagnosis of GVHD in situations where only distinctive clinical features of chronic GVHD are present, alternative diagnoses are entertained, clinical signs are confined to internal organs, or clinical assessment is obscured by prior changes. In these instances, histopathology should be viewed as essential for establishing diagnosis, especially if there are any atypical clinical features, confounding infections, or potential drug toxicity [15,16,17].

However, few studies have demonstrated the imaging characteristics of GI cGVHD forms, and these are limited to case reports [18,19,20,21,22,23,24].

The extraintestinal findings in this series of cases are in line with previous studies [28,29,30,31,32,33,34,35]. The most frequent finding was the comb sign. An important feature noted in this small series was the low presence of lymph nodes, associated with inflammation. The presence of ascites, which is a fairly frequent finding [27], was present in the acute phase in only two of our cases, probably because we also presented cases of acute, persistent, and chronic GVHD. Among the extraintestinal findings, the most frequent was dilation of the bile ducts. In particular, bile duct dilation is usually related to immune-mediated involvement of bile duct damage in GVHD. GVHD in fact causes atrophy of the bile ducts, lymphocyte infiltration, and necrosis [25,30,32]. Dilatation of the common bile duct (as in case 4) could be caused by the thickening of the walls of the duodenum and consequent stenosis of the papilla [30]. Even from this small series, it appears that the intestinal radiological findings of both acute (multifocal thickening, mucosal and stratified enhancement) and chronic (sub-stenotic thickening) GVHD, as well as the extraintestinal findings, can be very similar and overlap with those of IBD. However, in GI-GVHD, unlike in IBD such as Crohn’s disease, the inflammation is often limited to the mucosa, and the involvement of the intestinal loops is often more widespread; the loops have a serpiginous course and few reactive lymph nodes are present [27]. In Crohn’s disease, however, the inflammation is transmural and can be complicated with fistulas and abscesses, and the reparative fibrotic phenomena with phases of acuteness and remission are characteristic of the disease [43,44,45]. Furthermore, from a clinical point of view, diarrhea in GVHD is watery, whereas in Crohn’s disease it is often mucus-bloody, and skin involvement is much more common in GVHD [35]. Both IBD and GVHD can affect the biliary tract; however, while IBD such as ulcerative colitis can be complicated by primary sclerosing cholangitis, in which loss of the bile duct occurs due to progressive fibrosis, the damage to the bile duct is immune-mediated in GVHD [35]. Finally, there are marked differences in the mechanism and extent of tissue damage, which are clearly demonstrated by the intestinal histopathology of these diseases. Although both GVHD and IBD are characterized by damage to the intestinal barrier, in GVHD, the etiology is clearer, as the intestinal barrier is already damaged by the conditioning therapy of the pre-transplant phase. In IBD, etiology is multifactorial, and genetic, environmental, and immune factors are involved [43,44,45]. Histologically, GVHD is characterized by crypt cell apoptosis and glandular atrophy. The immune reaction is often poor, and both neutrophil infiltration and mucosal ulceration are present only in the most severe cases. In contrast, disease activity in IBD is defined by mucosal and intraepithelial neutrophils infiltrating and damaging the epithelium, forming crypt abscesses. Typically, these are accompanied by dense accumulations of lymphocytes, macrophages, and plasma cells within the lamina propria. Although epithelial apoptosis may be present in IBD, it is not a prominent histological feature in patient biopsies [35]. Furthermore, the glandular distortion that characterizes IBD, such as Crohn’s disease, is the result of repeated damage with reparative phenomena and is much more accentuated than in GVHD [35].

There are still few medium- and long-term follow-up studies that correlate the clinical characteristics of these patients with the radiological findings, most likely because the prognosis of these patients is often poor [34]. In the study by Brodoefel et al. [33], no differences were found in the CT patterns of acute and late-onset forms of GVHD. However, published studies are always based on small samples, and often the timing of the evaluation is not specified. The main limitation of CT imaging is that it can only detect macroscopic changes. These limitations could be overcome using MRI, which, being a multiparametric method, allows the study of finer alterations of the intestinal wall [28]. The main limitation of this retrospective evaluation is undoubtedly the limited number of patients. However, given the rarity of GVHD, this small series can provide important information for clinical practice.

## 4. Conclusions

Our small case series suggests that CECT evaluation may be useful for the management of HSCT patients with proven or suspected GI-GVHD. In our patients, some CT patterns appeared typical of GI damage associated with either acute, persistent forms of GVHD or chronic GVHD. Larger, systematic studies are needed to assess how radiological changes track with the unpredictable clinical course of GI-GVHD. This knowledge will improve our ability to make effective therapeutic decisions. The association of radiological findings with more recent biomarkers of GVHD (e.g., ST2, REG3a, and TIM3) or scoring systems (i.e., the MAGIC score) may also provide further data to establish how best to incorporate this relatively easy procedure into the algorithm of GI-GHVD diagnosis and management.

## Figures and Tables

**Table 1:** Summary of the clinical data for each case, including information on age; sex (male (M), female (F)); the underlying disease (UD) (acute lymphoid leukemia ALL, severe aplastic anemia (SAA), Hodgkin lymphoma (HL), and acute myeloid leukemia (AML)); stem cell source (SCS) (bone marrow (BM), peripheral blood (PB)); type of donor (TD) (matched unrelated donor (MUD)); time of onset of clinical symptoms (TCS); time of execution of CECT from HSCT (TCECT); clinical presentation (CP) (abdominal pain (AP), diarrhea (D), and small bowel occlusion (SBO)); type of GI-GVHD acute/chronic (a/c) (acute persistent (ap), acute classical (ac), chronic overlap (co)); clinical stadiation of GI-GVHD (STD GI-GVHD) on the basis of Glucksberg modified criteria (8,9); and outcome of patients (dead (D), alive (A)).

Cases	Age	Sex	HP	SCS	TD	TSO	TCECT	CP	GVHD-GI a/c	Cl. Std GI-GVHD	Outcome
***1***	28	F	AML	SB	haploidentical	35 days	155 days	AP + D	ap	III	D
***2***	51	M	HL	BM	haploidentical	15 days	20 days	AP + D	a	IV	D
***3***	47	F	ALL PH+	PB	Sibling	20 days	92 days 129 days	AP + D	ap	III	D
***4***	34	M	SAA	PB	Sibling	35 days	66 days 120 days (4 months) 240 days (8 months)	AP + D	ap	III	A
***5***	47	F	ALL PH+	BM	MUD 10/10	9 months	9 months	AP + SBO	co	III	D

**Table 2:** Summary of the intestinal, mesenteric, and extraintestinal findings of the five cases (FU: follow-up; P: present).

Cases	Ribbon Sign	Intestinal and Colic Fluid Distension	Intestinal Thickness and Enhancement	Colic Thickness and Enhancement	Mesenteric Findings	Extraintestinal Findings
**1**	P	P	Multisegmental and plurifocal parietal thickness with homogeneous mucosal enhancement on distal ileus	Multisegmental thickness with homogeneous mucosal enhancement	Mild diffuse comb sign and poor reactive mesenteric lymph node	Splenic infarct
**2**	P	P	Diffuse parietal thickness with stratified enhancement	Multisegmental thickness with homogeneous mucosal enhancement	Diffuse comb sign and poor reactive mesenteric lymph node	Mild gallbladder thickness and mild biliary tract dilatation
**3**	P	P	Multisegmental thickness with stratified enhancement at the terminal ileus (on CECT control at 92 days). The CECT control at 129 days showed persistence of mild parietal thickness at terminal ileus and with mucosal enhancement	Diffuse parietal thickness with stratified enhancement (On CECT control at 92 days) The CECT control at 129 days showed colic fluid distension with mild diffuse mucosal enhancement	Diffuse comb sign, mild ascites, and poor reactive mesenteric lymph node	Hepatomegaly and mild gallbladder thickness
**4**	P	P	Intestinal diffuse homogeneous thickness with homogeneous mucosal enhancement (on CECT control at 66 days after the HSCT); diffuse small bowel involvement with increased parietal thickness on CECT control at 120 days	Appearance of multisegmental thickness on sigma and descending colon and stratified enhancement on transverse colon on the CT FU at 120 days	Diffuse comb sign and poor reactive mesenteric lymph node	Mild gallbladder thickness and persistence of biliary tract dilatation on follow-up CT
**5**	P	P	Multisegmental thickness with stenosis on jejunal loops and SBO and segmental and focal thickness on the terminal ileus and appendix	Diffuse parietal thickness with stratified enhancement	Mild comb sign, poor reactive mesenteric lymph node, small air bubbles in the ascites	Small splenic areas of ischemia

## Data Availability

The data associated with the study were obtained from hospital rec-ords and can be made available on request.
